# Tumor suppression in mice lacking GABARAP, an Atg8/LC3 family member implicated in autophagy, is associated with alterations in cytokine secretion and cell death

**DOI:** 10.1038/cddis.2016.93

**Published:** 2016-04-28

**Authors:** F S Salah, M Ebbinghaus, V Y Muley, Z Zhou, K R D Al-Saadi, M Pacyna-Gengelbach, G A O'Sullivan, H Betz, R König, Z-Q Wang, R Bräuer, I Petersen

**Affiliations:** 1 Institute of Pathology, University Hospital – Friedrich Schiller University Jena, Ziegelmühlenweg 1, Jena D-07743, Germany; 2 Iraqi Centre for Cancer and Medical Genetics Research, Al-Mustansiriya University, Baghdad, Iraq; 3 Institute of Physiology 1, University Hospital – Friedrich Schiller University Jena, Teichgraben 8, Jena D-07743, Germany; 4 Leibniz Institute for Natural Product Research and Infection Biology, Hans Knöll Institute (HKI), Beutenbergstrasse 11, Jena D-07745, Germany; 5 Integrated Research and Treatment Center, Center for Sepsis Control and Care (CSCC), Jena University Hospital, Erlanger Allee 101, Jena D-07747, Germany; 6 Leibniz Institute for Age Research – Fritz Lipmann Institute (FLI), Beutenbergstrasse 11, Jena D-07745, Germany; 7 Institute of Pathology, University Medicine Berlin, Campus Charité Mitte, Berlin D-10098, Germany; 8 Department of Neurochemistry, Max-Planck Institute for Brain Research, Deutschordenstrasse 46, Frankfurt D-60528, Germany; 9 Max-Planck Institute for Medical Research, Jahnstrasse 29, Heidelberg D-69120, Germany; 10 Faculty of Biology and Pharmacy, Friedrich Schiller University Jena, Bachstrasse 18k, Jena D-07743, Germany

## Abstract

GABARAP belongs to an evolutionary highly conserved gene family that has a fundamental role in autophagy. There is ample evidence for a crosstalk between autophagy and apoptosis as well as the immune response. However, the molecular details for these interactions are not fully characterized. Here, we report that the ablation of murine GABARAP, a member of the Atg8/LC3 family that is central to autophagosome formation, suppresses the incidence of tumor formation mediated by the carcinogen DMBA and results in an enhancement of the immune response through increased secretion of IL-1*β*, IL-6, IL-2 and IFN-*γ* from stimulated macrophages and lymphocytes. In contrast, TGF-*β*1 was significantly reduced in the serum of these knockout mice. Further, DMBA treatment of these GABARAP knockout mice reduced the cellularity of the spleen and the growth of mammary glands through the induction of apoptosis. Gene expression profiling of mammary glands revealed significantly elevated levels of Xaf1, an apoptotic inducer and tumor-suppressor gene, in knockout mice. Furthermore, DMBA treatment triggered the upregulation of pro-apoptotic (Bid, Apaf1, Bax), cell death (Tnfrsf10b, Ripk1) and cell cycle inhibitor (Cdkn1a, Cdkn2c) genes in the mammary glands. Finally, tumor growth of B16 melanoma cells after subcutaneous inoculation was inhibited in GABARAP-deficient mice. Together, these data provide strong evidence for the involvement of GABARAP in tumorigenesis *in vivo* by delaying cell death and its associated immune-related response.

Gamma (*γ*)-aminobutyric acid type A receptor (GABA_A_R)-associated protein (GABARAP) was first identified as a trafficking molecule for GABA_A_Rs in neurons.^1^ We previously reported that GABARAP may function as a candidate tumor suppressor in breast cancer. Introduction of the gene into a breast cancer cell line reduced the growth rate and colony formation *in vitro* and suppressed tumorigenicity in nude mice. In addition, overexpression of the gene was associated with cytoplasmic vesicle formation,^2^ a finding supported by the fact that GABARAP has been shown to be involved in autophagy.^3^ Specifically, it was shown that GABARAP has a crucial role in the autophagic process through mediating membrane hemifusion and to be involved in the maturation of the autophagosome, a key component of autophagy machinery.^4, 5^ As autophagy is a fundamental mechanism for most cells, it is not surprising that the gene is ubiquitously expressed. Knockout (KO) of the gene, however, did not reveal an obvious pathological phenotype.^6^ This might be due to the fact that GABARAP belongs to a gene family. Its homolog in yeast, autophagy-related gene 8 (Atg8), is an essential gene and mutants die under starvation.^7^ In mammals, there are several Atg8 homologs grouped into two subfamilies: microtubule-associated protein-1 light chain 3 (LC3) and GABARAP, of which LC3 is the most well known as it is widely used to monitor autophagic activity.^8^ The fundamental importance of this gene family in mammals may also be derived from the fact that Atg8 is an ubiquitin-like protein. Thus it is possible that the Atg8 gene family may have a similar relevance for autophagy as ubiquitin has for proteasomal protein degradation.^9^

Autophagy is an intracellular pathway for bulk degradation of damaged proteins and organelles within the lysosome/vacuole to recycle building blocks for biosynthesis and cellular energy under conditions of stress.^10^ Its role in cancer is complex and controversial. It may act as a tumor-promoting as well as tumor-suppressive mechanism depending on the cellular context and the genetic background.^11^ In particular, there is a close relationship between autophagy and apoptosis and some of the molecular constituents in this interplay have already been identified. In most cases, autophagy delays or prevents apoptosis, but in a few circumstances, it may also assist cell death.^12^

Apart from apoptosis, autophagy is deeply integrated into tumor immunity,^13^ metabolism^14^ and the stress response.^15^ In addition, it has been identified as a potential target of cancer therapy.^16^ Interestingly, the disruption of Atgs has been associated with immunity and inflammation.^17^ Specifically, GABARAP KO was shown to increase the secretion of proinflammatory cytokines in the context of sepsis. KO mice were highly susceptible to mortality and revealed increased proinflammatory cytokine secretion through activation of the NOD-like receptor family, pyrin domain containing 3 inflammasome in two sepsis models.^18^ Tumor-promoting inflammation, avoiding immune destruction and resisting cell death have been identified as hallmarks for tumor initiation and progression.^19^ It has been proposed that cancer cell-associated autophagy has a key role in subverting anti-tumor immunity.^20^

The aim of our study was to use a GABARAP KO mouse model to evaluate the potential *in vivo* role of the gene in tumorigenesis and to clarify whether it acts as a tumor suppressor or enhancer. Our data confirm its relevance for tumor growth with respect to 7,12-dimethylbenz(a)anthracene (DMBA)-induced tumor development as well as syngenic tumor cell inoculation. Unlike our previous *in vitro* data, we find that GABARAP acts as tumor enhancer *in vivo*, which seems to be related to the inhibition of apoptosis and antitumor immune response.

## Results

### Reduction of tumor incidence in GABARAP KO mice

In order to investigate the potential roles of GABARAP in tumor incidence, female GABARAP KO (KO) and wild-type C57BL/6J (Wt) mice were treated with DMBA. Tumors arose in 14 of the 29 Wt mice (48.3%) within 35 weeks after the last dose of DMBA. In KO mice, tumor formation was significantly reduced (4 of 33; 12.1% *P*=0.002, Table 1). A variety of tumors developed in each group, for instance, mammary, skin and liver tumors as well as lymphomas. When only mammary tumors were considered, a difference between 21.4% (3 out of the 14 tumors in Wt mice) and 0% (0 of the 4 tumors in KO mice) was observed (Table 1).

**Figure 1:**
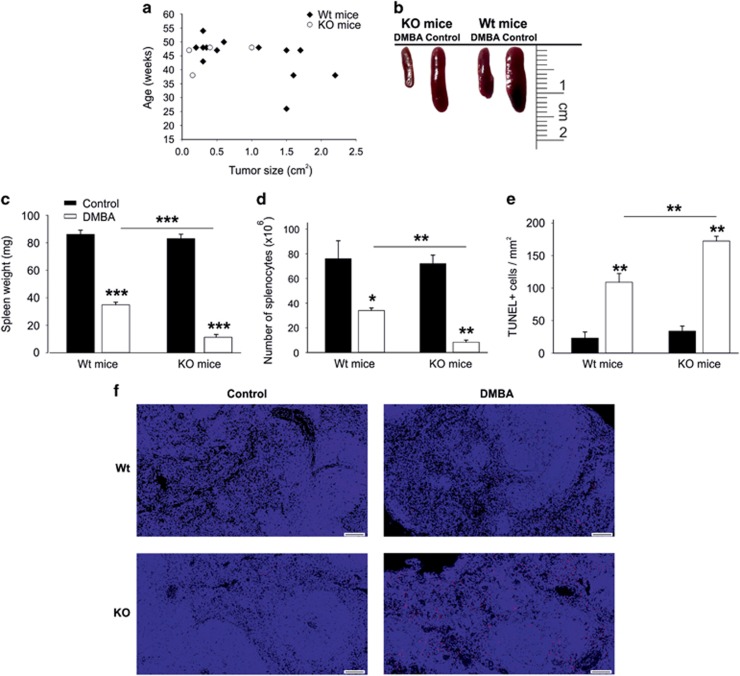
Impact of GABARAP deficiency on the mice upon DMBA treatment. (**a**) Size of all the tumors induced by DMBA in Wt and GABARAP KO mice. (**b**) Representative picture of the spleens removed from Wt and GABARAP KO mice at the same age (14 weeks) and treatment condition. (**c**) Spleen weights for vehicle-treated (control) and DMBA-treated mice (14 weeks). (**d**) Total number of splenocytes. The data were calculated using three mice per group and shown as means±S.E.M. (**e** and **f**), Quantitative analysis and immunofluorescence TUNEL staining of spleen sections. The data were calculated using 3–6 mice per group and shown as means±S.E.M. Scale bars=100 *μ*m. *P<0.05, **P<0.01, ***P<0.001

Large tumors (>1.5 cm^2^) were only found in Wt mice, whereas only small tumors (≤1 cm^2^) occurred in KO mice (Figure 1a).

### Enhanced sensitivity of GABARAP KO mice to DMBA induced immunotoxicity

In addition to mutagenicity, DMBA treatment elicits an immunotoxic effect mainly on splenic cells.^21^ In our model, both DMBA-treated groups reduced the spleen volume; however, KO mice showed a higher reduction in comparison with Wt mice after DMBA treatment (Figure 1b). Spleen weights of DMBA-treated Wt mice were decreased to about 40.5% (±0.7%) of vehicle-treated Wt mice (Figure 1c). However, a more substantial decrease was observed in spleen weights of DMBA-treated KO mice (13.6±1.8%, in comparison with vehicle-treated KO mice, Figure 1c).

Consistent with spleen weight, a significant decrease in the total number of splenocytes after DMBA treatment was seen in KO mice (11.6±2.4% of splenocytes from vehicle-treated KO mice) compared with Wt mice (44.7±2.8%, Figure 1d). Furthermore, fluorescence-activated cell sorting (FACS) analyses were carried out to detect the alterations in the cell populations of splenocytes. KO mice showed a substantial reduction in the number of macrophages, B cells, T cells (CD8^+^ and CD4^+^) and regulatory T cells (Tregs) compared with Wt mice after DMBA treatment (Supplementary Figures S1A–E). These results highlight the lack of a selective effect of DMBA on particular cell types in both KO and Wt mice. To assess whether the reduction in spleen size and weight was due to cell death, a terminal deoxynucleotidyl transferase dUTP nick-end labeling (TUNEL) assay was performed. TUNEL-stained spleen sections showed increased numbers of TUNEL-positive cells in the DMBA-treated groups. However, the number of TUNEL-positive cells was significantly increased in DMBA-treated KO mice compared with Wt counterparts (Figures 1e and f).

### Cytokine secretion from both macrophages and lymphocytes are elevated in GABARAP KO mice

**Figure 2:**
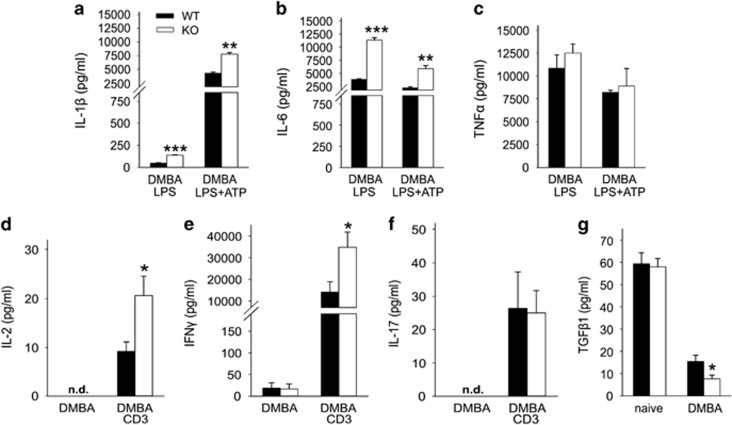
Cytokine secretion is enhanced in GABARAP KO mice. (**a**) IL-1*β*, (**b**) IL-6 and (**c**) TNF*α* were measured from the supernatants of LPS- and LPS+ATP-stimulated peritoneal macrophages after treatment of the mice with DMBA (*n*=3 per group). (**d**) IL-2, (**e**) IFN-*γ* and (**f**) IL-17 were measured in the supernatants of unstimulated and anti-CD3-stimulated (CD3) splenic lymphocyte after treatment of the mice with DMBA (*n*=6–11 per group). (**g**) TGF-*β*1 levels in the serum of Wt and GABARAP KO mice that had been untreated (naive) or treated with DMBA (*n*=6/8 per group). Data are representative of triplicate experiments and are shown as means±S.E.M. *P<0.05, **P<0.01, ***P<0.001

Many autophagic genes are known to have a role in immunity, and disruption of GABARAP gene has been reported to increase lethality and expression of interleukin (IL)-1*β* in two sepsis mouse models.^18^ Here we wanted to determine whether GABARAP deficiency has a role in cytokine secretion upon DMBA treatment. Lipopolysaccharide (LPS)/LPS+ATP (adenosine triphosphate)-stimulated macrophages from DMBA-treated KO mice secreted significantly more IL-1*β* and IL-6 than macrophages from Wt counterparts (Figures 2a and b). No significant differences were seen for tumor necrosis factor alpha (TNF*α*) secretion between both groups (Figure 2c).

DMBA-treated mice have been reported to be persistently immunosuppressed, mainly through suppression of IL-2.^22^ Surprisingly, in our model anti-CD3-stimulated lymphocytes from DMBA-treated KO mice produced significantly more IL-2 and interferon (IFN)-*γ* than stimulated lymphocytes from Wt counterparts (Figures 2d and e). No difference was seen for the secretion of IL-17 between both groups of mice (Figure 2f).

Interestingly, we found that the level of transforming growth factor beta 1 (TGF)-*β*1 was significantly reduced in the serum of DMBA-treated KO mice compared with Wt mice (Figure 2g). This is in line with the suppressive function of TGF-*β*1 towards T helper 1 cell (T_H_1)-derived cytokines, such as IL-2 and IFN-*γ*.^23^

### Cell death in mammary glands is promoted in mice lacking GABARAP

**Figure 3:**
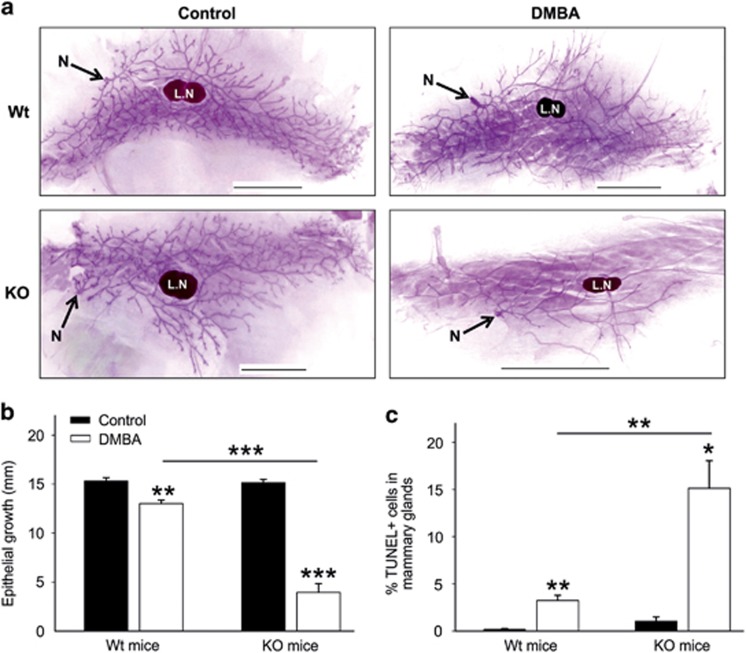
Elevated cell death in the mammary glands of GABARAP KO mice. (**a**) Whole mount analysis of mammary glands from 14-week-old female Wt and GABARAP KO mice, which were either vehicle-treated (control) or DMBA-treated. LN, lymph node, N, nipple; scale bars=5 mm. (**b**) Mammary epithelial cell growth in the mice as described in panel (**a**). The epithelial cell growth was determined by measuring the distance from the lymph node to the end of epithelial tree (in millimeter), using a ruler. Values are representative of 6–9 mice and are shown as means±S.E.M. (**c**) Quantification of the number of TUNEL positive cells within mammary gland sections. The data were calculated using 3–5 mice per group and shown as means±S.E.M. *P<0.05, **P<0.01, ***P<0.001

DMBA is highly carcinogenic when administered to adult female mice by oral gavage and especially relevant to study mouse mammary gland tumorigenesis.^24^ Therefore, we explored the mammary epithelial cell growth and ductal tree morphogenesis in our model. Mammary glands of both female KO and Wt mice at 14 weeks of age showed normal mammogenesis, and no obvious phenotypic difference in mammary epithelial cell growth was noticed (Figure 3a). Furthermore, the mammary glands of Wt mice treated with DMBA also showed phenotypically normal mammary epithelial trees compared with vehicle-treated Wt mice (Figure 3a). However, the mammary glands of DMBA-treated KO mice showed a massive reduction of epithelial cell outgrowth and ductal branching compared with vehicle-treated KO or DMBA-treated Wt mice (Figure 3a). Additionally, the lengths of the ductal outgrowths in DMBA-treated KO mice were significantly shorter than in all other groups of mice (Figure 3b). Moreover, TUNEL-stained mammary gland sections of DMBA-treated KO mice showed a significantly increased number of TUNEL-positive cells compared with the other groups (Figure 3c). Thus cellular death is highly elevated when these KO mice are exposed to the genotoxic carcinogen DMBA. This may explain the inhibition of tumor formation of mice after DMBA treatment.

### Increased expression of tumor-suppressor X-linked inhibitor of apoptosis protein (Xiap)-associated factor 1 (Xaf1) in GABARAP KO mice

**Figure 4:**
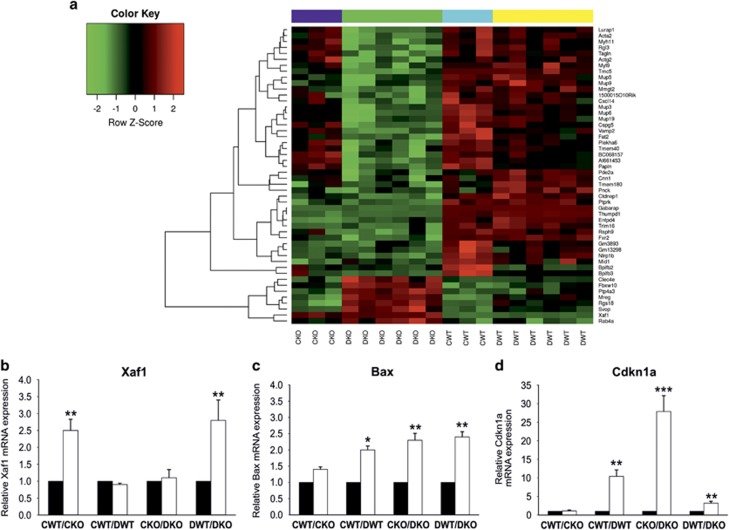
Influence of GABARAP ablation on the gene expression profile of mammary glands. (**a**) Heat map of differentially expressed genes in mammary glands of female Wt and GABARAP KO mice. A comparative analysis of control Wt (CWT) (*n*=3) and DMBA-treated Wt (DWT) (*n*=6) with their control KO (CKO) (*n*=3) and DMBA-treated KO (DKO) (*n*=6) counterparts identified 50 genes that were significantly altered. (**b**–**d**) Relative mRNA expression of Xaf1, Bax and Cdkn1a, respectively, in mammary glands of vehicle-treated (control) and DMBA-treated mice, as measured by quantitative reverse transcriptase PCR (qRT-PCR). Xaf1 expression was mainly influenced by the GABARAP genotype (**b**). Mammary glands from KO animals had elevated expression levels of Xaf1, a result that is consistent with the increased amount of apoptosis of these cells displayed. In contrast, Bax and Cdkn1a expression was not only affected by the genetic background of the animals (KO *versus* WT) but also by the DMBA treatment (**c**, **d**). The relative expression values of each gene were normalized to GAPDH for each sample. Values are mean±S.E.M. of 4–6 samples of each group. *P<0.05, **P<0.01, ***P<0.001

To investigate the potential cellular and molecular mechanisms by which mammary tumor development, branching and ductal outgrowth was suppressed in KO mice by DMBA treatment, microarray based gene expression profililing of the mammary glands was performed. The four experimental groups of mice chosen were: vehicle-treated Wt (CWT), vehicle-treated KO (CKO), DMBA-treated Wt (DWT), and DMBA-treated KO (DKO). From this data, a subset of genes whose expression was altered in a GABARAP-dependent manner between CWT and DWT was analyzed. The set of the most differentially expressed genes upon KO is shown in a heat map (Figure 4a). As expected, there was a pronounced difference in GABARAP expression between Wt and KO mice. In addition, the analysis revealed that Xaf1 gene was highly and differentially expressed in the mammary glands of KO mice (Figure 4a and Table 2). Xaf1 functions as a tumor suppressor, and its epigenetic silencing was associated with cancer development and progression.^25, 26, 27^ We additionally explored the relationship between GABARAP and Xaf1 expression by interrogating different expression databases. Interestingly, the levels of GABARAP were significantly higher when compared to Xaf1 in normal and malignant breast tissues as well as cancer cell lines from various organs (Supplementary Figures S2 and S3).

Xaf1 was originally identified as a novel negative regulator of Xiap.^28^ Interestingly, in our model Xiap shows a significant upregulation in Wt mice upon DMBA treatment, whereas DMBA-treated KO mice exhibited only modest downregulation of Xiap compared with vehicle-treated KO mice (Table 2). These findings suggest that Xaf1 upregulation in the mammary glands of KO mice contributes to the inhibition of tumor formation and in so doing enhance cell death induction upon DMBA treatment.

### Alteration in gene expression in mammary glands of DMBA-treated mice

We further analyzed the expression of genes controlling cell death, cell cycle, DNA replication and autophagy in the data obtained from our mammary gland microarray expression experiments. We found numerous genes that were differentially expressed upon DMBA treatment (Table 2). In DMBA-treated KO mice, pro-apoptotic (Bid, Apaf1), cell death (Tnfrsf10b, Ripk1) and cell cycle control (Cdkn2c) genes were upregulated compared with vehicle-treated KO mice. Bax, Siva1 and Cdkn1a were upregulated upon DMBA treatment in Wt as well as in KO mice, compared with their vehicle-treated counterparts. In Wt mice only, DMBA treatment had a positive effect on the expression of Cdc7, Cdk1 (genes encoding essential proteins of cell cycle control), E2f4, Tfdp2 and NF-κB1 (transcription factors). NF-κB1 had been reported to have a role in mammary tumorigenesis as an early event, which is induced by DMBA treatment.^24, 29^ Apart from those genes that were upregulated only a few genes were downregulated in the mammary glands of KO mice upon DMBA treatment (Stmn4, Tgfb3, E2f1). Overexpression of Siva1 had been found to inhibit stathmin (Stmn), an important regulatory protein of microtubule dynamics, leading to suppression of epithelial–mesenchymal transition and metastasis.^30^

Moreover, several autophagy genes were significantly upregulated in Wt and KO mice after DMBA treatment (Table 2). To further investigate the effect of DMBA on the autophagic response in GABARAP Wt and KO cells, we conducted western blotting analysis in mouse embryonic fibroblasts (MEFs). As depicted in Supplementary Figure S4, the conversion of soluble LC3A/B-I to lipid-bound LC3A/B-II is an indicator of autophagy induction in response to DMBA treatment. In addition, the upregulation of GABARAP and degradation of p62 in Wt MEFs indicates proper autophagic response while the impairment of autophagy in GABARAP-deficient MEFs is represented by the accumulation of p62 (Supplementary Figure S4).

To verify these results on a subset of genes, we performed qRT-PCR analysis using the same set of mRNAs that has been used for the oligo microarray chips. We found that the relative mRNA expression of Xaf1 was significantly upregulated in mammary glands of vehicle- and DMBA-treated KO mice in comparison to its Wt counterparts (Figure 4b). Consistent with the microarray data, we found that the expression levels of Bax and Cdkn1a were significantly upregulated in both groups of mice upon DMBA treatment. However, levels of Bax and Cdkn1a were significantly higher in the treated KO mice compared with their Wt counterparts (Figures 4c and d).

### GABARAP deficiency inhibits growth of inoculated tumor cells

**Figure 5:**
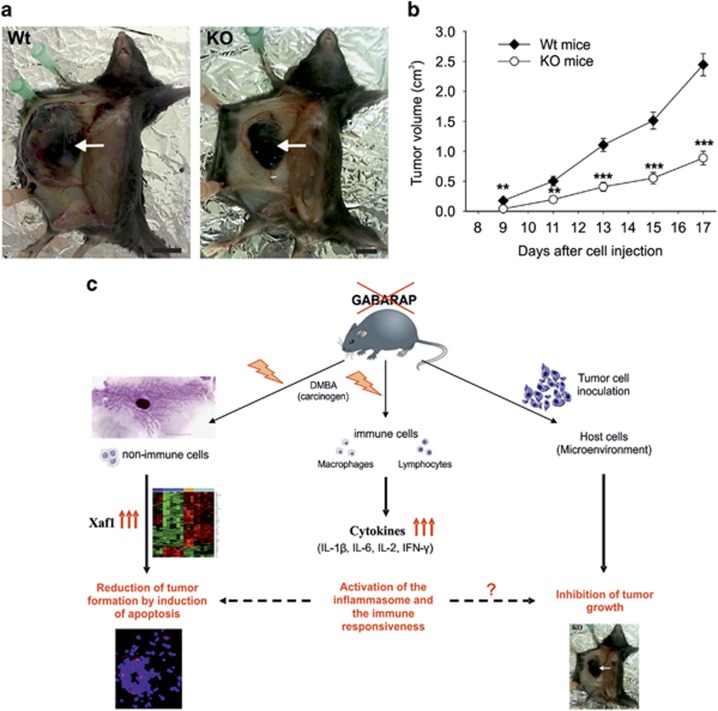
Inhibition of B16 melanoma cells growth in GABARAP KO mice and a working model to explain these results. (**a**) Still pictures taken from sacrificed Wt and GABARAP KO mice at the seventeenth day after the inoculation of 2.5 × 10^5^ B16 melanoma cells. Scale bars=10 mm. (**b**) Growth curve of B16 melanoma cells in Wt and KO mice. Values are representative of 12 mice and are shown as means±S.E.M. **P<0.01, ***P<0.001. (**c**) Working model summarizing the potential mechanisms of tumor suppression by GABARAP. The subjection of GABARAP KO mice to a genotoxic stress (DMBA) leads to the over and differential expression of the tumor-suppressor gene Xaf1 in non-immune cells, an effect that may trigger cell death events underlying the inhibition of tumor formation. GABARAP-deficient immune cells produce elevated levels of several cytokines under genotoxic stress, which could suppress tumor formation and/or may trigger cell death. Similar mechanisms in the derangement of immune signaling may be involved in growth suppression seen in the GABARAP KO tumor graft model. This prediction requires further investigation in order to clarify the role of GABARAP removal in the reduction of inoculated tumor cell growth

We also analyzed whether the genetic background of the recipient may affect the tumor growth of inoculated B16 melanoma cells. All the Wt mice presented palpable tumors at the ninth day of cell injection compared with 66.7% of KO mice (Figures 5a and b). The tumor growth increased constantly in both groups, but the tumor volume was significantly decreased in KO mice compared with Wt mice over the whole period of observation (Figure 5b). This result suggests that intact GABARAP function in the host animal is advantageous for the growth of the inoculated syngenic melanoma cells.

## Discussion

In this study, we examined the potential role of GABARAP in tumor formation induced by the chemical carcinogen DMBA. We demonstrated that the ablation of GABARAP expression significantly diminished tumor incidence. GABARAP has a fundamental role in autophagosome formation, a key structure of the autophagy machinery.^5^ Contradictory results have been reported for the roles of specific autophagy genes in neoplasia. Despite that Beclin1 having been demonstrated as having a tumor-suppression function for autophagy,^31^ KO of Atg5, Atg7 and FIP200, other essential autophagy proteins, failed to initiate malignant tumor development *in vivo*.^32, 33^ Based on our knowledge, this is the first report providing *in vivo* evidence for the involvement of GABARAP in tumorigenesis.

It has been previously reported that DMBA treatment resulted in decreased spleen weight and increased splenocyte death in mice.^21^ In agreement with these results, DMBA-treated Wt mice showed a reduction in spleen size, weight and cell number. Importantly, these effects were more pronounced in KO mice and were accompanied by increased numbers of TUNEL-positive splenocytes. Thus GABARAP deficiency promoted cellular death after genotoxic stress by DMBA, which is consistent with the role of its homolog Atg8 in yeast and the concept that autophagy blockade may enhance cellular death under stress conditions.^7, 12^

The immune response has an essential role in delineating tumor outgrowth and progression. Cytokine secretion can trigger cell signaling toward antitumor effects and/or cell death. This cell to cell communication relies on a panel of cytokines and an accurate assignment of effector cells. We found that macrophages from DMBA-treated KO mice significantly boosted the secretion of IL-1*β* and IL-6 upon stimulation. It has been reported that DMBA treatment causes oxidative stress and mitochondrial dysfunction^34^ and that autophagy (mitophagy) is required for mitochondrial maintenance.^15^ Accumulation of damaged mitochondria could activate the inflammasome and thereby increase proinflammatory cytokine secretion.^17, 18^ Therefore, we propose that GABARAP deficiency under a genotoxic stress of DMBA may activate the inflammasome in macrophages by preventing the removal of damaged mitochondria, which are responsible for the increased production of proinflammatory cytokines. In addition, IL-1*β* was shown to be engaged in different cellular signaling pathways. Roy *et al.*^35^ demonstrated that high concentrations of IL-1*β* induced apoptosis, whereas moderate or low levels kept the same cells normal or stimulated their growth.

It is known that DMBA treatment results in suppression of the immune response for prolonged periods of time through inhibition of lymphocyte activity, mainly by suppressing IL-2 production. Thus DMBA stimulates tumorigenesis via two mechanisms. First, by inducing DNA mutations, and second, by downregulating the immune response that is relevant for cancer cell outgrowth after tumor initiation.^22^ The response in GABARAP KO mice seems to be different. Lymphocytes of DMBA-treated KO mice produced significantly higher levels of IL-2 and IFN-*γ*. These results indicated that an immune enhancement of GABARAP-deficient immunocytes by DMBA treatment had occurred. Several reports have shown the efficacy of IL-2 and IFN-*γ* in antitumor immunity by promoting innate and adaptive immune responses.^36, 37^ De Palma *et al.*^38^ and Wei *et al.*^33^ showed effective inhibition of PyMT-driven tumor growth and metastasis through upregulation of IFN target genes. Furthermore, Atg5 knockdown in human melanoma cells enhanced IL-6 production and proliferation of anti-tumorigenic CD8^+^IFN-*γ*^+^ and CD4^+^IFN-*γ*^+^ T cells.^39^

To obtain a better insight into the mechanism underlying IL-2 and IFN-*γ* elevation, we analyzed TGF-*β*1 secretion in the serum. TGF-*β*1 was significantly reduced in DMBA-treated KO mice. Tregs have the ability to produce TGF-*β*.^23^ Our FACS analyses showed that there was a substantial reduction of Tregs in the spleen of DMBA-treated KO mice, which could be responsible for TGF-*β*1 depression. Thus our findings are consistent with previous observations demonstrating that TGF-*β* has a suppressive function towards T_H_1 cells,^23^ suggesting that this is the reason for the enhancement of IL-2 and IFN-*γ* production. Altogether, our results revealed that the KO of GABARAP has a considerable impact on the immunity of mice through enhancing the secretion of regulatory cytokines upon carcinogen treatment.

Our results demonstrated that mammary tumors were less frequent in KO mice after DMBA treatment. This was accompanied by a highly significant reduction of mammary epithelial growth. Moreover, the gene expression profile revealed significant upregulation of several cell death genes. Xaf1 was found to be differentially expressed in the mammary glands of KO mice, irrespective of whether these mice were treated with DMBA or not. This gene has been extensively studied in the past decade and has been characterized as an apoptosis-inducer and tumor-suppressor gene. Extremely low or undetectable Xaf1 expression is a frequent event in several cancer cell lines,^25^ as well as in human cancer tissues.^26, 27^ The restoration of Xaf1 expression induced cancer cell apoptosis, cell cycle arrest and inhibited tumor growth in various types of cancers, as well as resulting in an increased cell sensitivity to drug-induced apoptosis. ^26, 27, 40^

Xaf1 was originally identified as a binding partner and a novel negative regulator of apoptosis-inhibitor protein, Xiap thereby acting itself as an apoptosis inducer.^28^ Xiap functions through its binding to TNF receptor-associated factors and caspase family and, thereby, inhibits apoptosis.^28^ We found that Xiap was significantly upregulated in the mammary glands of Wt mice upon DMBA treatment, whereas the mammary glands of DMBA-treated KO mice exhibited a modest downregulation. Xaf1 has been reported to induce cell death through multiple mechanisms. Straszewski-Chavez *et al.*^41^ showed that Xaf1 re-localized to mitochondria in response to TNF-related apoptosis-inducing ligand and promoted translocation of Bax into mitochondria and cytochrome *C* release and thereby induced apoptosis. Consistent with these observations, our results revealed an enhancement of the pro-apoptotic genes Bid, Apaf1 and Bax, which are involved in the intrinsic activation of apoptosis by mitochondrial outer membrane permeabilization.^12, 42^ Activation of the mitochondrial apoptotic pathway by Xaf1 was associated with dramatically enhanced TNF*α* expression.^42^ We did not detect any difference in TNF*α* expression in stimulated macrophages. However, we found that Tnfrsf10b and Ripk1, both being TNF receptors, were significantly upregulated in the mammary glands of DMBA-treated KO mice, suggesting that the TNF*α* signaling pathway may have been affected.

Our results have elucidated that autophagy genes are upregulated after DMBA treatment. Indeed, cell exposure to genotoxic carcinogens trigger several cellular processes to bypass the damage induced by such chemicals. Autophagy is one of these processes that is stimulated in order to cope under times of stress.^15^ As autophagy is dependent on GABARAP, its deficiency may weaken the maintenance of tissue homeostasis leading to increased incidence of cell death. The interplay between autophagy and apoptosis has only partially been defined to date.^12^ The differential expression of GABARAP and Xaf1 may suggest that these genes constitute part of a molecular switch in the decision of a cell to undergo autophagy or apoptosis. The nature of how this switch in gene expression operates remains a mystery. Interestingly, Xaf1 has been recognized as an IFN-stimulated gene and its expression has been promoted by both IFN and TNF*α*.^43^ IFN-induced Xaf1 expression occurs either through its interaction with the IFN regulatory factor 1-(IRF-1) binding element (IRF-E) or demethylation of CpG sites within the Xaf1 promoter.^44, 45^ The influence of IFN on Xaf1 has been shown to occur through the JAK-STAT and JNK pathways in a transcription dependent manner through the recruitment of IRF-1.^45^ Recently, Qiu *et al.*^46^ demonstrated an IRF-1-enhanced Xaf1 gene dependent activation within glomerular mesangial cells of a rat nephritis model. Therefore, Xaf1 might represent a mediator for apoptosis that is induced by cytokines.^45^ Based on our findings, we propose that cytokines are involved in the enhancement of Xaf1 expression, thus pointing to an indirect mechanism of Xaf1 regulation upon GABARAP removal.

Finally, our results provide evidence for the role of autophagy in anti-tumor immunity, as we showed that B16 melanoma cells' growth in GABARAP KO mice is reduced. Further evidence for this link comes from work done by Noman *et al.*,^47^ who inoculated Beclin1 knockdown B16 melanoma cells in a syngenic allograft tumor mouse model. In line with our results, a decrease in tumor formation was observed and the autophagy dysfunction was linked to the dysregulation of antigen-specific T-cell lysis.^47^ To our knowledge, our investigation represents the first study that investigated the role of tumor cell growth by inoculating syngenic tumor cells into autophagy-deficient mice. As the GABARAP-deficient immune cells displayed an alteration in cytokine secretion that was able to boost the anti-tumor immune response, we would like to propose that this effect may have influenced the growth of the B16 melanoma cells by similar mechanisms that had been proposed by Noman *et al.*,^47^ that is, anti-tumor immunity. At least, a cell autonomous interplay between autophagy and apoptosis mediated by GABARAP could not explain this effect by itself. The precise role of tumor growth reduction needs to be clarified through further studies. For example, the role of microenvironment factors that represent an essential demand for tumor growth *in vivo*, needs to be investigated.

In summary, we have identified a novel role of GABARAP in tumorigenesis by using a mouse model and show that ablation of GABARAP *in vivo* inhibits tumor initiation and progression through enhancement of both antitumor immunity and cell death signaling, as summarized in a working model (Figure 5c). The challenge for future investigations will be to explore the molecular mechanisms of GABARAP in the regulation of gene expression and immunomodulation upon cellular genotoxic insult.

## Materials and Methods

### Mice and DMBA treatment

Wt C57BL/6J mice were purchased from Charles River (Sulzfeld, Germany). GABARAP KO mice were established from a colony initially maintained at the Max Planck Institute for Brain Research (Frankfurt, Germany). The strain background of the GABARAP-deficient mice is the C57BL/6J strain. As previously reported, GABARAP KO mice were phenotypically indistinguishable from Wt mice and showed no detectable changes in gross anatomy.^6^ DMBA (Sigma-Aldrich, Taufkirchen, Germany) was dissolved in sesame oil (5 mg/ml). Mice at 6–8 weeks of age received 6 weekly doses of 1 mg DMBA/mouse by oral gavage. Vehicle-treated (control) groups received just sesame oil. The mice were monitored weekly for palpable tumor formation. Tumor specimens were fixed and then stained with hematoxylin and eosin. All animal experiments were approved by the local government commission for animal protection (No. 02-018/08 and 02-007/13).

### TUNEL assay

Tissue sections were deparaffinized, transferred to antigen retrieval buffer, heated in a microwave until boiling and kept at sub-boiling temperature for 10 min. After cooling, sections were washed with PBS and reacted with terminal deoxynucleotidyl transferase mixture (Fermentas, St. Leon-Rot, Germany) according to the manufacturer's instructions. Subsequently, after washing, sections were incubated with Streptavidin-Cy3 (Sigma-Aldrich) for 2 h. Then sections were washed again and mounted with coverslips and a DAPI-containing mounting medium (ProLong Gold, Life Technologies, Darmstadt, Germany). Tissue section images were acquired using a microscope (Virtual microscope BX61VS, Olympus, Tokyo, Japan).

### FACS analysis

For the analysis of cell surface markers, single-cell suspensions from the spleens were prepared 1 week after the last dose of DMBA. Cells were permeabilized by 0.5% (w/v) saponin in PBS (containing 0.25% (w/v) bovine serum albumin and 0.02% (w/v) sodium azide). Splenocytes (macrophages, B cells, CD4^+^ T cells and CD8^+^ T cells) were analyzed by staining with an antibody solution containing anti-CD11b-Alexa Fluor 700, anti-B220-Pacific Blue, anti-CD4-Dye647 and anti-CD8-PE/Cy7 (all obtained from eBioscience, Frankfurt, Germany). Tregs were analyzed by staining with anti-CD4-APC/Cy7 and anti-CD25-Dye647 and subsequent incubation with FoxP3 Fixation/Permeabilization working solution (all obtained from eBioscience). Then cells were stained with anti-FoxP3-FITC (eBioscience). All samples were measured using the flow cytometer BD LSR II (BD Biosciences, San Jose, CA, USA).

### Cytokine analysis

Cytokines were quantified in single-cell suspensions from the spleens and peritoneal macrophage cultures prepared 1 week after the last dose of DMBA. As there is almost no basal release of cytokines under *in vitro* conditions, lymphocytes (2.5 × 10^5^ cells/ml) were cultured in RPMI medium (Biochrom, Berlin, Germany) containing 10% (v/v) FCS (Biochrom) for 48 h with 1 *μ*g/ml plate-bound anti-CD3e antibodies (eBioscience) for overall T-cell receptor stimulation. Macrophages (1.5x10^5^ cells/ml) were stimulated with 1 *μ*g/ml LPS (Sigma-Aldrich) (4 h) and 5 mM ATP (InvivoGen, Toulouse, France) (1 h) in RPMI medium containing 10% (v/v) FCS. Cytokines were measured in the supernatants using standard sandwich ELISA procedures as previously described.^48^ Primary and biotin-labeled secondary antibodies for IL-1*β*, IL-6, TNF*α*, IL-2 and IFN*γ* were purchased from BD Biosciences (Heidelberg, Germany) and antibodies for IL-17 from R&D Systems (Wiesbaden, Germany). For quantification, recombinant cytokines were used as standard. Absorbance was measured at 492 nm. TGF*-β*1 was determined at the same time in the serum using an ELISA Kit (R&D Systems) according to the manufacturer's instructions.

### Whole-mount analysis of the mammary glands

The fourth abdominal mammary gland was dissected from the pelt and spread on a glass slide and then submerged into a mixture of absolute ethanol: glacial acetic acid, 3 : 1 (v/v) for 3 h. Subsequently, the glands were washed with 70% (v/v) ethanol and rinsed in distilled water. The glands were stained with aluminium carmine (Fluka, Bassersdorf, Switzerland) overnight. Then the slides were rinsed with 70, 90 and 100% (v/v) ethanol and transferred to xylene for clearing and mounted with aqueous mounting medium (Permount, Fisher Scientific, Pittsburgh, PA, USA). Analysis of mammary epithelial growth was performed by measuring the distance from the lymph node to the end of epithelial tree using a ruler, according to de Assis *et al.*^49^

### Microarray gene expression profile

Abdominal mammary glands were isolated and directly fixed in 5% (w/v) neutral-buffered formalin and subsequently paraffin blocks were constructed. Microarray workflow was carried out by Miltenyi Biotec GmbH (Bergisch Gladbach, Germany), using the Agilent Whole Mouse Genome oligo microarrays 8 × 60K chips (Agilent Technologies, Santa Clara, CA, USA) according to standard protocols of formalin-fixed paraffin-embedded (FFPE) samples. Briefly, RNA isolation was performed using the Absolutely RNA FFPE Kit (Stratagene, Agilent Technologies, Santa Clara, CA, USA) and cDNA was obtained using the Complete TransPlex Whole Transcriptome Amplification Kit WTA2 (Sigma, St. Louis, MO, USA). DNA samples were labeled using the Genomic DNA ULS Labeling Kit (Agilent Technologies) to produce Cy3-labeled DNA. The hybridization procedure was performed overnight according to the Agilent Gene Expression FFPE Workflow protocol using the Agilent Gene Expression Hybridization Kit and Agilent CGHblock. Fluorescence signals were detected and processed using Agilent's Microarray Scanner System and Feature Extraction Software (Agilent Technologies). Microarray image files were processed using Limma package for the R language.^50^ Background correction was performed and arrays were normalized using loess method. The probes were selected based on having expression intensities that were above background at least in three samples. Expression levels were averaged for genes having more than one probe set. Raw *P*-values for differential gene expression were derived from moderated *t*-statistic^50^ and subsequently corrected using the Benjamin and Hochberg approach.^51^ The gene expression differences with *P*-values ≤0.05 were considered as differentially expressed.

### Real-time RT-PCR analysis

The same sets of RNA that were used in microarray analysis were used for reverse transcription. Total RNA was reverse transcribed into cDNA using a QuantiTect Reverse Transcription Kit (Qiagen, Hilden, Germany) according to the manufacturer's instructions. Real-time RT-PCR was performed on the Rotor-Gene 6000 system (Qiagen) using FastStart Universal SYBR Green Master (Roche, Mannheim, Germany). Twenty-five nanograms of RNA were used for PCR amplification with the following primer pairs for Xaf1: 5′-TCCAAGTGTGCAGGAACTG-3′ (forward), 5′-CAACTTCCATGTGCTCTTTCATC-3′ (reverse), Bax: 5′-TTGGAGATGAACTGGACAGC-3′ (forward), 5′-CAGTTGAAGTTGCCATCAGC-3′ (reverse), Cdkn1a: 5′-AGGCCCAGTACTTCCTCTGC-3′ (forward), 5′-CAATCTGCGCTTGGAGTGATA-3′ (reverse), and glyceraldehyde-3-phosphate dehydrogenase (GAPDH): 5′-CACACCGACCTTCACCATTTT-3′ (forward), 5′-GAGACAGCCGCATCTTCTTGT-3′ (reverse). The relative expression value of each gene was normalized to GAPDH for each sample.

### Tumor inoculation

The murine B16 melanoma cell line was kindly provided by the Institute of Pathology, Charité Hospital (Berlin, Germany). Cells were cultured in RPMI 1640 medium (Biochrom) supplemented with 10% (v/v) FCS (Biochrom). For subcutaneous inoculation into the left flank (100 *μ*l), the cells were adjusted to a concentration of 2.5 × 10^6^ cells/ml in ice-cold PBS. After 1 week, the area where the inoculated cells had been injected were monitored daily for tumor growth. Tumor volume was measured every other day using a caliper.

### Statistical analysis

Data are expressed as mean±S.D./S.E.M. as indicated. The significance of tumor incidence between Wt and GABARAP KO mice was calculated using a Fisher's exact test. In the other experiments, differences between groups were calculated using the two-tailed Student's *t*-test for unpaired values. The normal distribution of the values was checked using the Kolmogorov–Smirnov test. Statistical significance was calculated by the SPSS software package (v.16.0, SPSS, Chicago, IL, USA), *P*-values <0.05 were considered as being significant.

## Figures and Tables

**Table 1:** Tumor types and incidence in C57BL/6 J wild-type (Wt) and GABARAP KO (KO) mice after DMBA treatment

**Tumor type**	**C57BL/6J (Wt)**	**GABARAP KO (KO)**
**DMBA treatment**	***n*=29**	***n*=33**
Mammary	3	—
Skin	7	2
Lymphoma	2	—
Liver	—	1
Undifferentiated tumors	2	1
Total	14 (48.3%)	4 (12.1%)**

**Significant difference between two groups (*P*<0.01) calculated by Fisher's exact test

**Table 2:** List of differentially expressed genes in mammary glands of Wt and KO mice

**Gene**	**Full name**	**CWT *versus* CKO**	**CWT *versus* DWT**	**CKO *versus* DKO**	**DWT *versus* DKO**	**Function**
GABARAP	Gamma-aminobutyric acid receptor-associated protein	−4.05^a^	0.2	0.09	−4.16^a^	Autophagy
Xaf1	XIAP-associated factor 1	3.25^a^	0.22	0.36	3.39^a^	Apoptosis
Xiap	X-linked inhibitor of apoptosis	0.51	0.86^a^	0.05	−0.31	Apoptosis
Bid	BH3 interacting domain death agonist	0.16	0.53	0.76^a^	0.39	Apoptosis
Apaf1	Apoptotic peptidase activating factor 1	−0.33	0.08	0.59^a^	0.18	Apoptosis
Bax	BCL2-associated X protein	0.03	1.79^a^	2.03^a^	0.27	Apoptosis
Tnfrsf10b	Tumor necrosis factor receptor superfamily, member 10b	−0.02	0.62	0.89^a^	0.25	Cell death
Ripk1	Receptor (TNFRSF)-interacting serine-threonine kinase1	0.24	0.29	0.53^a^	0.48	Cell death
Siva1	Apoptosis-inducing factor	0.08	1.3^a^	1.79^a^	0.57	Apoptosis
Stmn4	Stathmin-like 4	−0.01	−0.51	−1.14^a^	−0.65	Microtubule destabilizer
Il1r1	Interleukin 1 receptor, type I	−0.41	0.08	0.95^a^	0.45	Cytokine receptor
Cdkn1a	Cyclin-dependent kinase inhibitor 1A (P21)	−0.1	3.08^a^	3.73^a^	0.55	Cell cycle control
Cdkn2c	Cyclin-dependent kinase inhibitor 2C (p18)	−0.16	0.58	0.86^a^	0.12	Cell cycle control
Rbx1	Ring-box 1	0.17	0.49^a^	0.3	−0.02	Cell cycle control
Cdc7	Cell division cycle 7	0.29	0.5^a^	0.23	0.02	Cell cycle control
Cdk1	Cyclin-dependent kinase 1	0.77	1.06^a^	0.35	0.07	Cell cycle control
Tgfb3	Transforming growth factor, beta 3	−0.19	−0.51	−0.86^a^	−0.55	DNA replication
NF-κB1	Nuclear factor of kappa light polypeptide gene enhancer in B-cell 1	0.34	0.71^a^	0.6	0.23	Transcription factor
Smad2	SMAD family member 2	0.42	0.67^a^	0.38	0.13	Transcription factor
E2f1	E2F transcription factor 1	−0.22	−0.9^a^	−0.62^a^	0.07	Transcription factor
E2f4	E2F transcription factor 4	0.71	1.24^a^	0.76	0.23	Transcription factor
Tfdp2	Transcription factor Dp 2	0.17	0.68^a^	0.47	−0.04	Transcription factor
GABARAPL2	GABARAP-like 2	0.09	0.65^a^	0.62^a^	0.06	Autophagy
Atg12	Autophagy-related 12	−0.02	0.53^a^	0.62^a^	0.07	Autophagy
Prkaa1	Protein kinase, AMP-activated, alpha 1 catalytic subunit	0.2	0.79^a^	0.7^a^	0.11	Autophagy
Atg3	Autophagy-related 3	0.1	0.79^a^	1.08^a^	0.4	Autophagy
Atg4b	Autophagy-related 4B, cysteine peptidase	−0.57	−0.65^a^	−0.15	−0.07	Autophagy
Atg5	Autophagy-related 5	0.33	0.5^a^	0.28	0.11	Autophagy

Abbreviations: CKO, control (vehicle-treated) GABARAP KO (*n*=3); CWT, control (vehicle-treated) wild-type (*n*=3); DKO, DMBA-treated GABARAP KO (*n*=6); DWT, DMBA-treated wild-type (*n*=6). The values in table represent the logarithmic fold change for each gene

a Significant expression difference between two groups (*P*≤0.05) calculated by moderated *t*-statistic

## Abbreviations

Atg: autophagy-related gene
ATP: adenosine triphosphate
CD: cluster of differentiation
DMBA: 7,12-dimethylbenz(a)anthracene
FFPE: formalin-fixed paraffin-embedded
GABARAP: gamma (*γ*)-aminobutyric acid type A receptor (GABA_A_R)-associated protein
GAPDH: glyceraldehyde-3-phosphate dehydrogenase
IL: interleukin
IFN-*γ*: interferon gamma
Irf: interferon regulatory factor
KO: knockout
LC3: microtubule-associated protein-1 light chain 3
LPS: lipopolysaccharide
TGF- *β*1: transforming growth factor beta 1
TNF*α*: tumor necrosis factor alpha
Tregs: regulatory T cells
TUNEL: terminal deoxynucleotidyl transferase dUTP nick end labeling assay
Wt: wild-type
Xaf1: X-linked inhibitor of apoptosis protein associated factor 1

## Supplementary Material

Supplementary Figure Legends

Supplementary Figure1

Supplementary Figure2

Supplementary Figure3

Supplementary Figure4
