# Supplementary material for: Tumor suppression in mice lacking GABARAP, an Atg8/LC3 family member implicated in autophagy, is associated with alterations in cytokine secretion and cell death
Source: Cell Death Dis. 2016 Apr 28;7(4):e2205–. doi: 10.1038/cddis.2016.93 (PMC4855672; doi:10.1038/cddis.2016.93)
Supplement: Supplementary Figure Legends [file cddis201693x1.docx]

**Figure S1.** Alterations of splenocyte populations after DMBA treatment. Cell surface marker expression of: (**A**) macrophages, (**B**) B cells, (**C and D**) T cells (CD8^+^ and CD4^+^) and (**E**) FoxP3^+^ CD25^+^ T cells (Tregs) in the spleen of control and DMBA-treated mice. Data are representative of 2-3 mice per group and were shown with means ± SEM. (*) *P* < 0.05.

**Figure S2.** Expression distribution of GABARAP and Xaf1 in normal and malignant breast tissues. The RNA-seq expression data was obtained from The Cancer Genome Atlas (TCGA) database 1100 breast tissue samples. The data was analyzed for detection of outlier samples, and associated clinical metadata. The 905 samples belonging to normal breast tissue (n=88), triple negative tumours (n=121) and non-triple negative tumours (n=696) based on clinical data were used for plotting GABARAP and Xaf1 expression distribution. GABARAP expression level is significantly higher in all groups. *P*-values were calculated using Mann–Whitney U paired test. (*) *P* < 0.05.

**Figure S3.** Expression distribution of GABARAP and Xaf1 in various cancer cell lines. The normalized expression data was downloaded from The Cancer Cell Line Encyclopedia (CCLE) database. Cell lines were grouped according to their primary site of origin. Primary sites having more than 20 cell lines therein were selected for plotting expression of GABARAP and Xaf1. The GABARAP expression level is significantly higher in all primary sites. *P*-values were calculated using Mann–Whitney U paired test. (*) *P* < 0.05.

**Figure S4.** Induction of autophagy in MEFs exposed to DMBA. Mouse embryonic fibroblasts (MEFs) isolation was carried out according to the protocols of culture of animal cells (Freshney, 2005). Wild-type and GABARAP KO MEFs cultured in 10 cm petri dishes and treated with 100 nM DMBA for 24 and 48 h. The cells were lyzed and analyzed by western blotting using antibodies against LC3A/B (Cell Signaling, MA, USA), GABARAP (abcam, Cambridge, UK), p62 (Novus Biologicals, CO, USA), and β-Actin (Millipore, Darmstadt, Germany). As shown in the Figure, induction of autophagy was detected in MEFs after treatment with DMBA. The conversion of soluble LC3A/B-I to lipid bound LC3A/B-II was shown as an indicator of autophagy induction as well as upregulation of GABARAP in wild-type MEFs and accumulation of p62 protein in GABARAP-deficient MEFs.

*Freshney RI. Culture of animal cells: A manual of basic technique. (5^th^ edition) John Wiley & Sons: New York, USA, 2005, pp 176-199.*
