# Supplementary figures and images for: Tumor suppression in mice lacking GABARAP, an Atg8/LC3 family member implicated in autophagy, is associated with alterations in cytokine secretion and cell death
Source: Cell Death Dis. 2016 Apr 28;7(4):e2205–. doi: 10.1038/cddis.2016.93 (PMC4855672; doi:10.1038/cddis.2016.93)

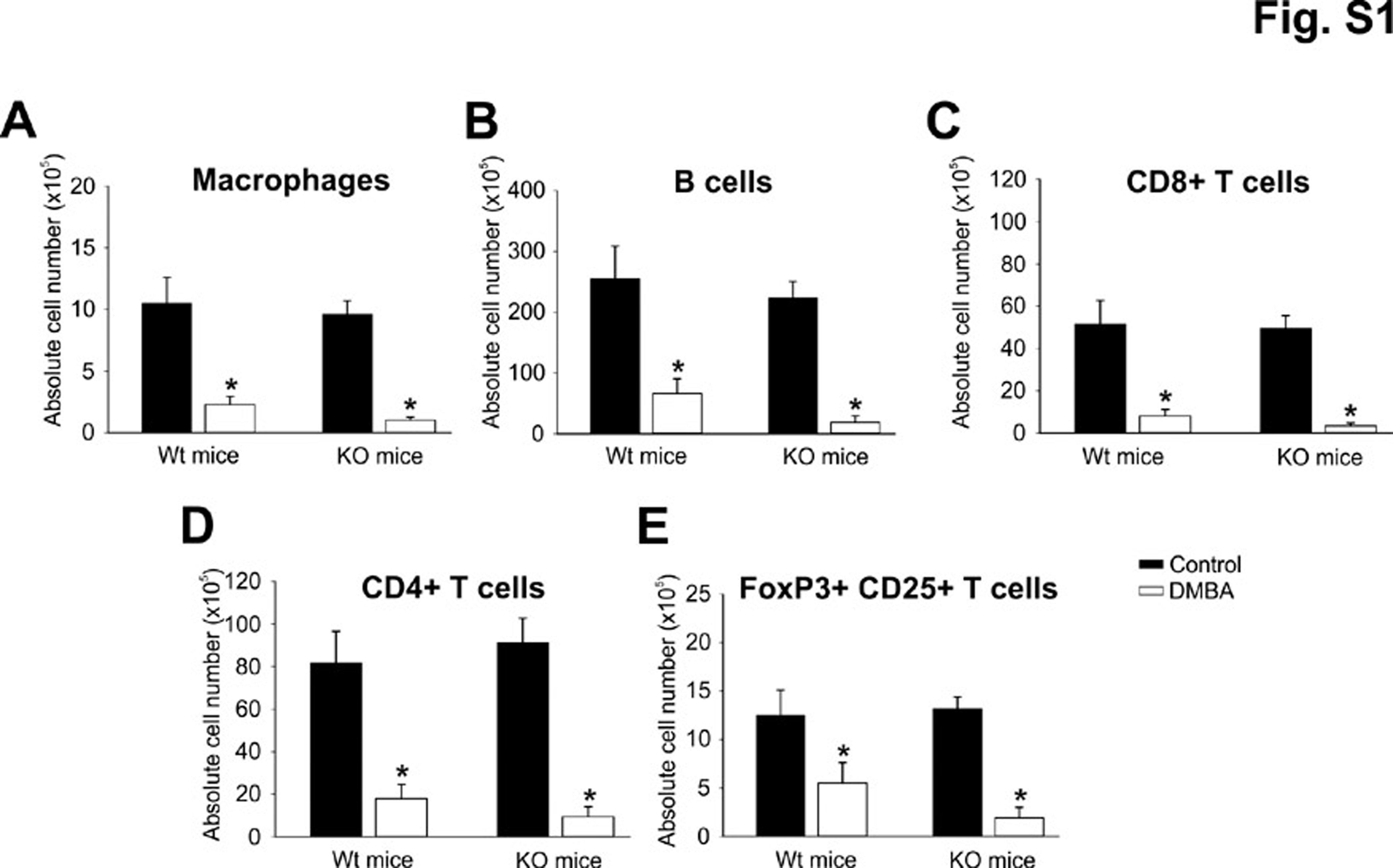

Supplement: Supplementary Figure1 [file cddis201693x2.tif]

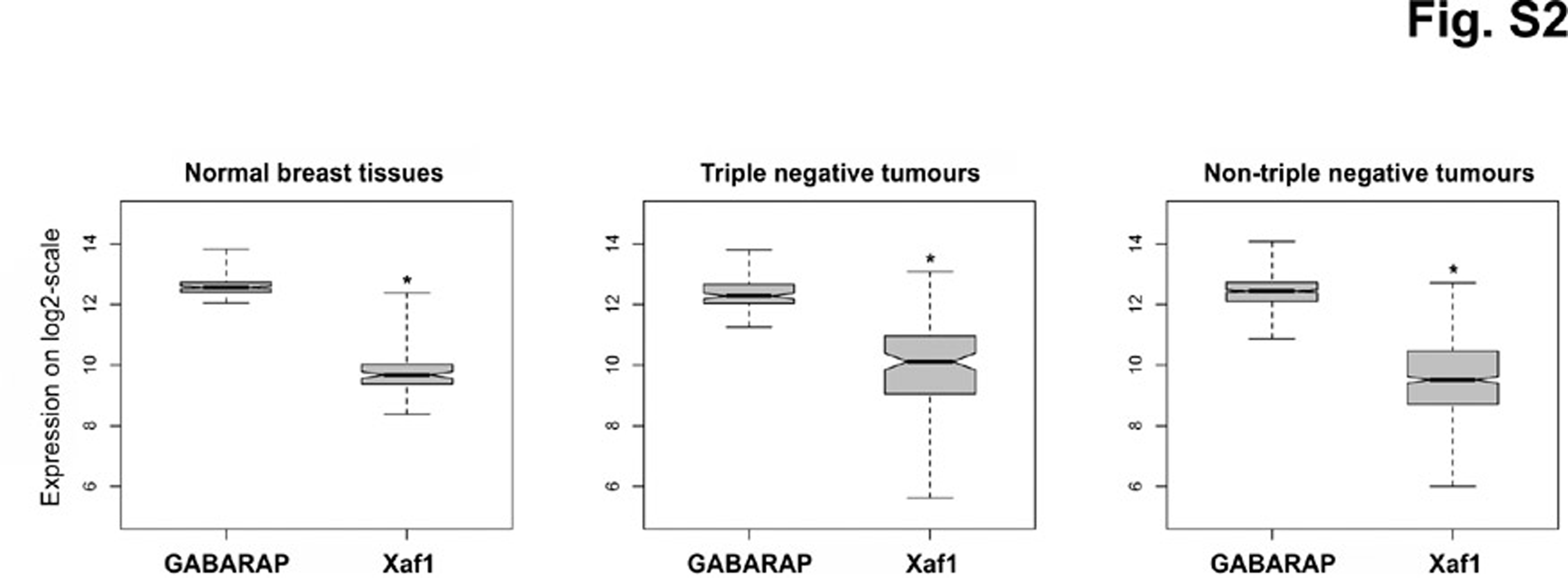

Supplement: Supplementary Figure2 [file cddis201693x3.tif]

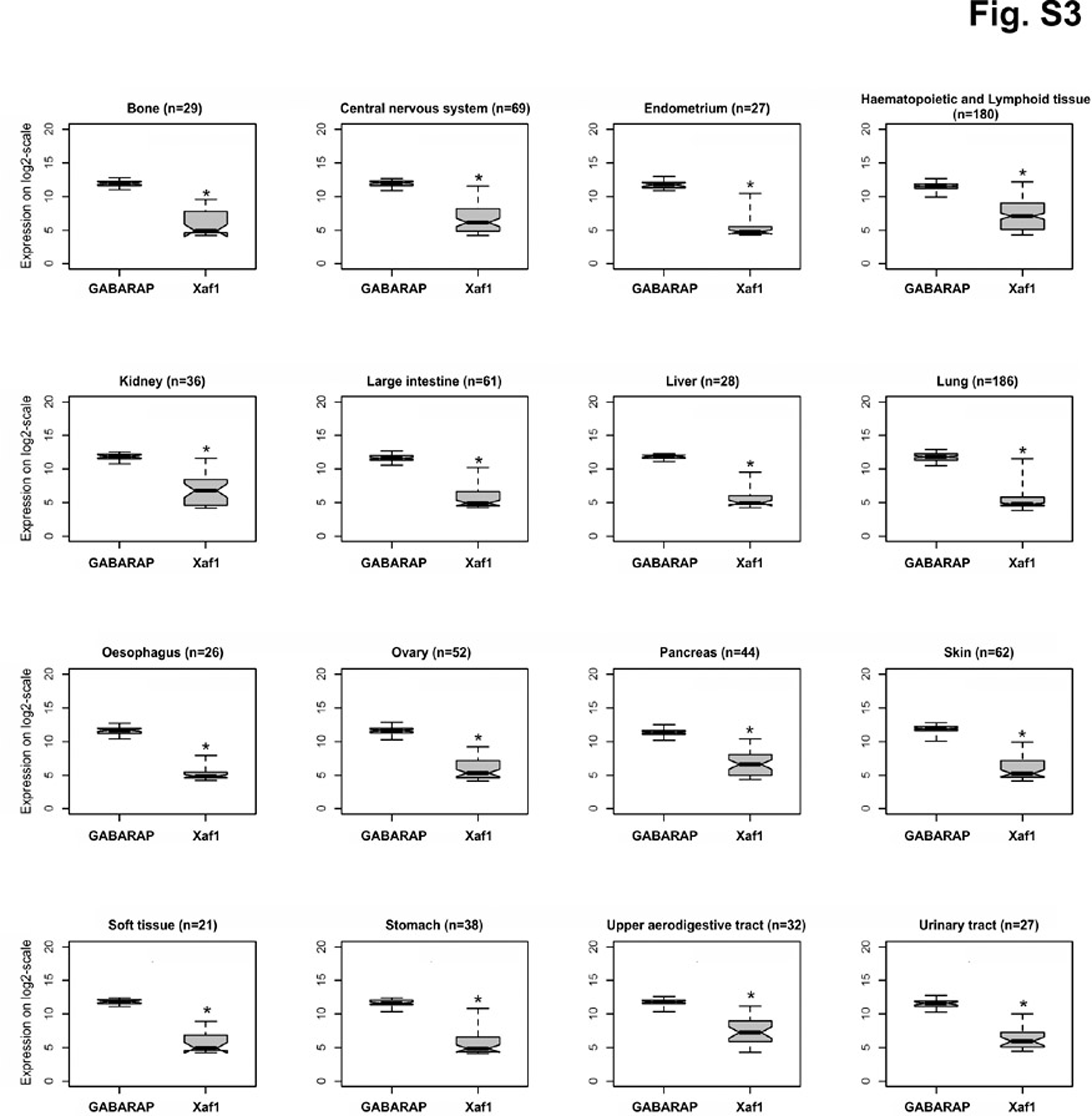

Supplement: Supplementary Figure3 [file cddis201693x4.tif]

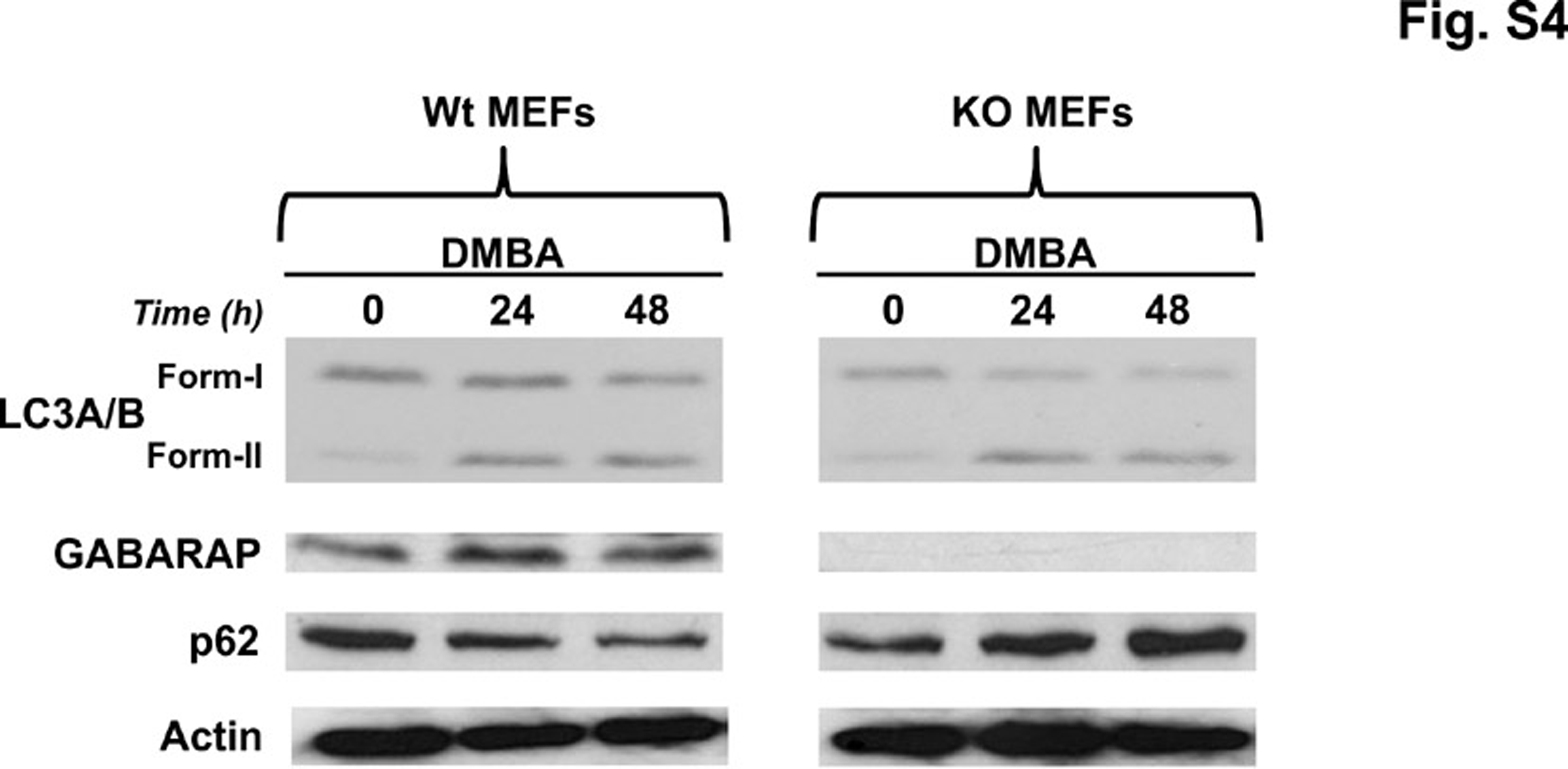

Supplement: Supplementary Figure4 [file cddis201693x5.tif]
